# MuSC cultivation for cultured meat production: recent advances, current limitations, and future directions

**DOI:** 10.1007/s44463-026-00108-2

**Published:** 2026-10-03

**Authors:** Eun Ju Lee, Khurshid Ahmad, Sibhghatulla Shaikh, Jeong Ho Lim, Syed Sayeed Ahmad, Min- Soo Seo, Inho Choi

**Affiliations:** 1 Department of Medical Biotechnology, Yeungnam University, 38541 Gyeongsan, Korea, Republic of; 2 Research Institute of Cell Culture, Yeungnam University, 38541 Gyeongsan, Korea, Republic of; 3 Department of Veterinary Surgery, College of Veterinary Medicine, Kyungpook National University, 41566 Daegu, Korea, Republic of

**Keywords:** Cultured meat, Muscle stem cells (MuSCs), Culture media optimization, Artificial intelligence (AI), Bioprocess engineering, Food technology

## Abstract

Cultured meat is being investigated as a potential alternative to conventional livestock production by cultivating animal cells under controlled conditions to reproduce key components and structural characteristics of meat. Muscle stem cells (MuSCs), which proliferate, differentiate, and fuse into multinucleated myofibers, are the principal cell source for muscle tissue engineering. However, commercial-scale production remains limited by the restricted proliferative and myogenic potential of MuSCs during extended in vitro expansion, the lack of cost-effective and food-safe culture media, and the challenge of recreating the complex architecture of native skeletal muscle. Addressing these limitations requires integrated advances in serum-free media optimization, identification of functional components via in silico and artificial intelligence (AI)-driven approaches, biomaterial scaffold engineering, and scalable bioprocessing. Current evidence suggests that no single technological breakthrough is sufficient; rather, convergence across cell biology, computational media design, and bioprocess engineering is essential. This review provides a comprehensive framework for addressing these scientific and technical barriers and discusses strategies that may support the development of safe, affordable, and structurally relevant cultured meat products.

## Introduction

Cultured meat is an emerging concept that currently lacks a universally accepted academic definition as its scope continues to evolve. For clarity, we define cultured meat as “a food product derived from the in vitro cultivation of animal cells that mimics the composition and structure of conventional meat.” Here, conventional meat refers to “tissue obtained directly from specific parts of animals, including livestock, for human consumption.” Conventional meat consists predominantly of skeletal muscle (SKM) along with associated connective tissues, blood vessels, nerves, and adipose tissue, constituting approximately 30–50% of total livestock body weight (Zao et al., 2026). Although the ultimate goal of cultured meat is to replicate these complex biological tissues, achieving true structural and compositional parity remains challenging due to the intricate architecture of SKM.

SKM, the primary component of meat, is attached to bones and facilitates movement through contraction and relaxation. Beyond mechanical movement, SKM stores energy as glycogen and performs vital physiological functions, including maintaining body temperature and protecting internal organs. The myofiber is the fundamental structural and functional unit of SKM. In native muscle, muscle satellite cells (MuSCs; also known as muscle stem cells) reside between the sarcolemma and the basement membrane. In response to external signals, MuSCs proliferate, fuse, and mature to form multinucleated myofibers. This process, termed myogenesis, is regulated by a complex cascade of growth factors, cytokines, and hormones that drive stage-specific transcription factor expression (Dumont et al., 2015; Sousa-Victor et al., 2022). Myofibers comprise numerous myofibrils composed of repeating sarcomeres, the primary contractile units consisting of actin and myosin. However, like other primary cells, MuSCs are prone to phenotypic drift, experiencing reduced proliferative and myogenic capacities during extended in vitro passage. Consequently, current research focuses on recapitulating key aspects of in vivo myogenesis to sustain MuSC proliferation, differentiation, and structural maturation in vitro.

Animal cell culture entails maintaining cells in an artificial environment optimized for viability, proliferation, or differentiation. Initially applied primarily in the life sciences and medicine, cell culture technology has expanded significantly to encompass biopharmaceutical production of monoclonal antibodies, vaccines, recombinant proteins, stem cell therapies, and safety testing (Butler, 2005; Gowder, 2017). While cultured meat relies on techniques adapted from biopharmaceutical production, it requires unique considerations; the final product must be food-safe, scalable, and produced at a fraction of the cost of biopharmaceuticals.

The essential elements required for cell culture include cell sources, culture media, and appropriate bioprocessing facilities. MuSCs are the most widely utilized cell source for cultured meat research. Because harvest strategies, storage protocols, and culture conditions directly influence cellular yield and function (Lanzoni et al., 2022; Martins et al., 2024), establishing robust and scalable culture systems is critical. Standard basal media containing glucose as the main carbon and energy source, along with amino acids, vitamins, and inorganic salts are typically supplemented with fetal bovine serum (FBS), growth factors, and antibiotics. Media used for cultured meat production must also be safe for human consumption and cost-effective. However, replacing FBS and high-cost recombinant supplements with safe, food-compatible, and economically viable alternatives remains a primary bottleneck for commercialization.

Furthermore, biomaterial scaffolds capable of mimicking the unique three-dimensional (3D) architecture of SKM are essential for guiding cell alignment and tissue maturation (Hong & Do, 2024; Shang et al., 2025; Zo et al., 2025). Because cultured meat is intended for human consumption, production systems must support long-term cultivation under food-grade or Good Manufacturing Practice (GMP) compliant standards while mitigating microbial contamination risks.

**Fig. 1:**
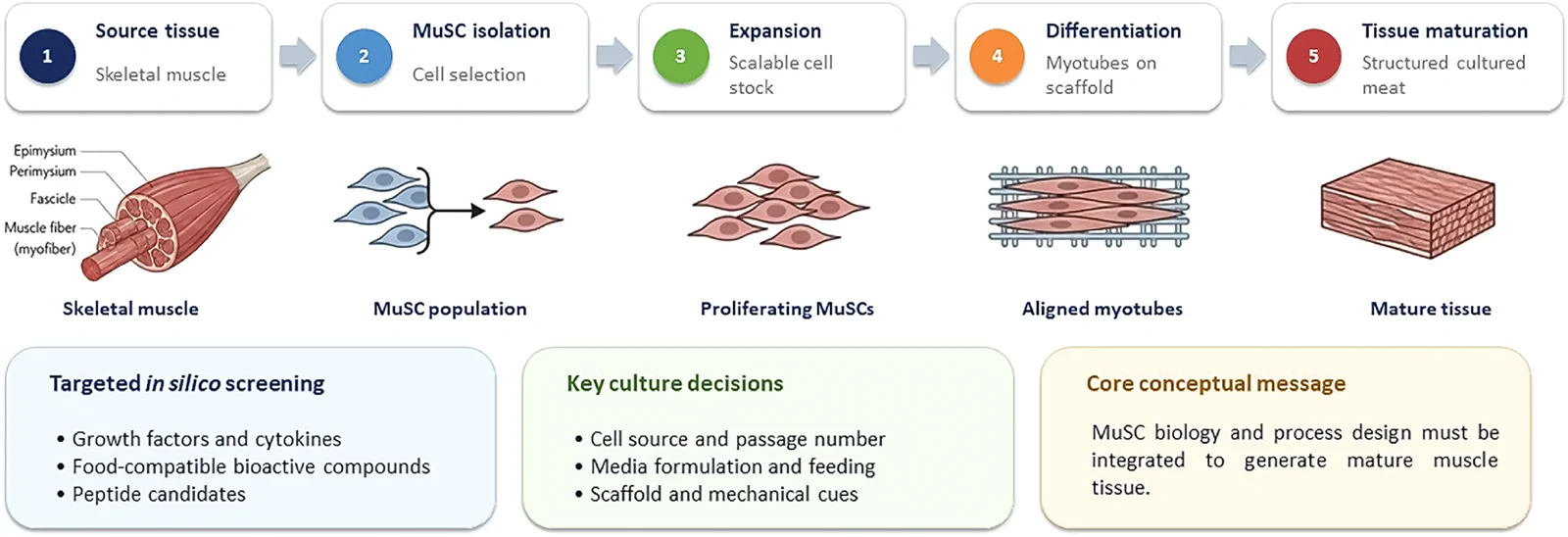
The role of MuSCs in cultured meat production. Schematic overview of the major stages of MuSC-based cultured meat production, from skeletal muscle tissue as the cell source, through MuSC isolation, expansion, myogenic differentiation, and tissue maturation. The process highlights the central role of MuSC proliferation and differentiation in generating aligned myotubes and mature muscle tissue. The lower panels summarize key factors that can be targeted through in silico screening, including growth factors and cytokines, food-compatible bioactive compounds, and peptide candidates, as well as key culture parameters such as cell source and passage number, medium formulation, and scaffold and mechanical cues. Integration of MuSC biology with optimized culture conditions and tissue-engineering strategies is essential for the efficient production of structured and mature cultured meat

In this review, we provide a comprehensive overview of recent advances in MuSC-based cultivation strategies for cultured meat production, focusing on methods that enhance cell expansion, myogenic differentiation, and overall process efficiency. We discuss the major technical hurdles limiting commercial feasibility, including finite proliferative capacity during extended passage, dependence on serum-containing media, and the lack of cost-effective, food-grade culture systems. Finally, we highlight emerging approaches that offer promising pathways forward, including serum-free media formulation and artificial intelligence (AI)-assisted identification of functional media components. By integrating insights from MuSC biology, computational media design, and tissue engineering, this review provides a strategic roadmap toward scalable and economically viable cultured meat production (Fig. 1).

## Methodology

This review focuses on studies examining MuSC isolation, proliferation, differentiation, and applications in cultured meat production. To provide a comprehensive overview of MuSC-based cultured meat systems, a structured literature search and narrative synthesis were conducted.

A structured search was performed using PubMed, Scopus, and Web of Science databases, covering publications from 1968 to 2026, with the final search conducted in August 2026. The search strategy combined terms such as “muscle stem cells,” “muscle satellite cells,” “myogenic differentiation,” and “cultured meat” using Boolean operators (AND, OR). Articles were selected based on their direct relevance to MuSC biology, cultivation strategies, culture media development, biomaterial scaffolds, and challenges associated with scalability and cost-effectiveness. Studies were considered eligible if they addressed MuSC isolation, expansion, differentiation, culture media formulation, or bioprocessing applications for cultured meat, whereas non-relevant studies or those lacking explicit experimental data were excluded. Literature was initially screened by title and abstract, followed by a full-text assessment. Additionally, recent advances involving in silico modeling and AI-assisted strategies for culture media optimization and process efficiency were incorporated.

This methodological approach facilitated the identification of recent technical advancements, ongoing limitations, and strategic pathways to enhance the scalability, efficiency, and economic feasibility of MuSC-based cultured meat production. Because this study was designed as a structured narrative review rather than a formal systematic review, a study-quality or risk-of-bias assessment was not performed.

## MuSCs as a cell source for cultured meat production

The selection of an appropriate cell source is a critical prerequisite for efficient and scalable cultured meat production (Post, 2012). Several cell types have been explored as potential candidates, including pluripotent stem cells, adult progenitor cells, mesenchymal stromal cells (MSCs), immortalized myogenic cell lines, and reprogrammed somatic cells. Pluripotent stem cells, including embryonic stem cells (ESCs) and induced pluripotent stem cells, possess robust self-renewal capacity and broad differentiation potential; however, their directed differentiation into stable myogenic lineages requires complex, tightly controlled protocols, and their regulatory approval for food applications remains to be established (Post, 2012; Domagała et al., 2026). MSCs derived from various tissues exhibit multipotent differentiation capacity and relatively high expansion potential, but they are not intrinsically committed to the myogenic lineage, resulting in lower and more variable efficiency in generating mature muscle fibers compared to muscle-specific progenitor cells (Pittenger et al., 1999; Okamura et al., 2018). Fibroblast-derived myogenic cells and immortalized cell lines offer alternative strategies by enabling the use of readily expandable starting cells or extended passage lifespans. Nevertheless, challenges related to incomplete lineage conversion, long-term phenotypic stability, and food-grade regulatory safety remain significant hurdles (Davis et al., 1987; Stout et al., 2023; Domagała et al., 2026).

In contrast, MuSCs are resident adult stem cells located within SKM tissue that are intrinsically committed to the myogenic lineage. This lineage commitment enables rapid and efficient differentiation into multinucleated muscle fibers under straightforward induction conditions (Yin et al., 2013; Ding et al., 2018). Furthermore, MuSCs can be directly isolated from livestock SKM tissue, providing direct biological relevance for reconstructing muscle structures that closely resemble conventional meat (Table 1). Although MuSCs possess a finite proliferative lifespan during extended in vitro expansion, their high myogenic fidelity and physiological relevance make them the primary and most practical cell source for current cultured meat research (Ding et al., 2018; Messmer et al., 2022).

**Table 1:** Comparison of cell sources for cultured meat production

Cell source	Origin	Key features	Advantages for cultured meat production	Limitations	References
ESCs	Inner cell mass of early-stage embryos	Pluripotent; extensive self-renewal	High proliferative potential and theoretical capacity to generate myogenic and adipogenic lineages	Ethical concerns; complex directed differentiation; residual undifferentiated-cell and regulatory concerns	Domagała et al. (2026); Post (2012); Thomson et al. (1998)
iPSCs	Reprogrammed adult somatic cells, including fibroblasts	Pluripotent; potentially extensive self-renewal	Avoids embryo-derived cell sourcing; potentially scalable source of myogenic and adipogenic progenitors	Technically demanding reprogramming and differentiation; possible genomic/epigenetic abnormalities; food-safety and regulatory considerations	Domagała et al. (2026); Post (2012); Takahashi and Yamanaka (2006)
MuSCs	Adult skeletal muscle; beneath the basal lamina of myofibers	Muscle-resident adult stem cells intrinsically committed to the myogenic lineage	High myogenic differentiation and fusion capacity; robust myotube formation; high physiological relevance for skeletal-muscle-like products	Finite proliferative lifespan; loss of stemness/myogenicity during prolonged expansion; donor variation; isolation and culture optimization required	Ding et al. (2018); Stout et al. (2022); Yin et al. (2013)
Immortalized myogenic cells	Satellite cells, primary myoblasts, or other myogenic progenitors subjected to immortalization	Extended proliferation; myogenic differentiation depends on cell line and immortalization strategy	More consistent and scalable supply than primary satellite cells; reduced donor variation; useful for bioprocess development	Immortalization-associated genomic changes and genetic modification may require careful safety, regulatory, and consumer-acceptance assessment; differentiation may differ from primary cells	Domagała et al. (2026); Stout et al. (2023)
MSCs	Bone marrow, adipose tissue, umbilical tissue, or other mesenchymal tissues	Multipotent stromal cells; differentiation depends on tissue source and induction conditions	Accessible and expandable; may contribute adipogenic, stromal, or connective-tissue components in multicellular products	Not intrinsically myogenic; myogenic differentiation is protocol- and source-dependent and generally less established than for satellite cells	Domagała et al. (2026); Okamura et al. (2018); Pittenger et al. (1999)
Fibroblast-derived myogenic cells/reprogrammed fibroblasts	Adult fibroblasts converted through transcription-factor-mediated reprogramming or transdifferentiation	Abundant, readily expandable starting cells; induced phenotype depends on conversion method	Potentially accessible alternative that may reduce reliance on muscle biopsies	Incomplete/heterogeneous conversion; variable phenotypic stability and terminal differentiation; suitability for food-scale production remains to be validated	Davis et al. (1987); Domagała et al. (2026)
Other adult progenitor cells (e.g., adipose-derived stromal/stem cells or pericytes)	Adipose tissue, vascular niches, and other adult tissues	Adult progenitor populations with source-specific differentiation potential	May support fat, connective-tissue, and other structural components that improve product characteristics	Lower myogenic specificity than satellite cells; heterogeneous and condition-dependent differentiation outcomes	Domagała et al. (2026); Zuk et al. (2002)

## SKM architecture and myogenesis

### SKM architecture and extracellular matrix (ECM)–cell interactions

SKM is the principal component of the musculoskeletal system and the primary edible tissue targeted in muscle-based cultured meat production. Structurally, SKM consists of multinucleated myofibers organized into hierarchical bundles, interspersed with adipose tissue, connective tissue, blood vessels, and resident stem and stromal cell populations. Myofibers contain repeating sarcomere units composed mainly of actin and myosin, which drive muscle contraction (Mukund & Subramaniam, 2020). In conventional meat, sensory texture and quality are dictated by the density, alignment, and composition of these muscle fibers and their associated extracellular matrix (ECM). Replicating these structural features in cultured meat is essential, as the spatial organization of muscle fibers and ECM proteins directly govern tissue architecture, mechanical properties, and ultimate mouthfeel.

The connective tissue network is organized into three distinct structural layers - the endomysium, perimysium, and epimysium - which contain diverse ECM proteins that provide essential structural and biochemical cues to surrounding muscle cells. Importantly, the ECM is not merely a passive structural scaffold; it constitutes a critical component of the MuSC niche, actively regulating cell adhesion, quiescence, activation, proliferation, and differentiation. For instance, collagen VI is an essential component of the native MuSC niche, regulating local biochemical properties to sustain satellite cell self-renewal and muscle regeneration (Urciuolo et al., 2013). These ECM–cell interactions are directly applicable to cultured meat systems, in which engineered biomaterials and scaffolds are deployed to provide cell adhesion, mechanical support, and spatial alignment for myogenic cells.

However, in cultured meat manufacturing, increasing myofiber size alone does not guarantee superior tissue quality. In engineered 3D muscle constructs, fiber diameter, cell density, construct thickness, and scaffold architecture must be optimized simultaneously. As tissue thickness increases, diffusion distances for oxygen and nutrients expand significantly, creating metabolic constraints. Thus, the design of cultured muscle constructs must balance myofiber maturation and structural alignment with efficient mass transfer and nutrient transport. Achieving true structural and textural fidelity thus requires precise orchestration of ECM–cell interactions, myofiber alignment, 3D architecture, and nutrient delivery.

### MuSCs and myogenesis

Cultured meat production relies on isolating and expanding primary myogenic cells, followed by their directed differentiation into mature SKM and adipose tissues. Among potential cell sources, MuSCs are particularly advantageous because they are resident adult stem cells responsible for muscle growth, repair, and regeneration. Positioned within a specialized niche between the myofiber sarcolemma and the basal lamina, quiescent MuSCs express key markers such as Pax7 (Urciuolo et al., 2013). Upon activation by environmental cues, MuSCs enter the cell cycle, proliferate, and undergo myogenic differentiation and fusion to form multinucleated myotubes and mature myofibers, while a subpopulation undergoes self-renewal to replenish the stem cell pool. Balancing this self-renewal, proliferation, and differentiation dynamics is a central requirement for cultured meat production: extensive proliferation is needed to accumulate cellular biomass, whereas efficient differentiation and fusion are required to achieve structural and textural fidelity.

Myogenesis is strictly orchestrated by a sequential network of myogenic regulatory factors (MRFs), including myogenic factor 5 (MYF5), myogenic differentiation 1 (MYOD), myogenin (MYOG), and myogenic regulatory factor 4 (MRF4), which serve as crucial molecular indicators for evaluating the myogenic status of expanding cells (Zammit, 2017). Although immortalized rodent myoblast lines such as C2C12 (Yaffe, 1968) and L6 (Yaffe & Saxel, 1977) are widely used to study fundamental myogenic mechanisms, their physiological responses often diverge from those of primary livestock cells. This distinction is critical when translating laboratory findings to cultured meat systems, as species- and cell-type–specific variations significantly affect proliferation kinetics and differentiation potential. Furthermore, primary livestock MuSCs face major technical hurdles during scale-up; prolonged in vitro expansion frequently induces cellular senescence, resulting in progressive loss of myogenic capacity (Olenic et al. 2025a). Developing targeted strategies to delay replicative senescence while preserving stemness is therefore essential for sustained cell expansion.

Importantly, MuSC performance varies depending on livestock species, breed, donor age and sex, and muscle anatomical origin. Therefore, researchers should report metrics such as population doubling time, proliferation rate, differentiation kinetics, and fusion efficiency as quantitative performance benchmarks. For instance, Kim et al. (2024b) showed that MuSCs isolated from Hanwoo steers exhibited higher proliferation rates and shorter doubling times than those from Hanwoo cows, along with distinct marker expression profiles and differentiation kinetics. Muscle of origin also influences cell performance because baseline variations in fiber-type composition, microenvironmental niche architecture, and local metabolic properties differ across SKMs. To ensure reproducibility and enable meaningful cross-study benchmarking, future cultured meat studies should routinely report species, breed, donor characteristics, tissue origin, passage number, proliferation rate or doubling time, and fusion metrics. Standardizing these quantitative descriptors will accelerate the identification of robust cell lines and guide species- and breed-specific optimization of culture media, growth-factor cocktails, and bioprocessing parameters (Muller & Matsusaki, 2025).

## Regulatory factors in myogenesis

### Growth factors, cytokines, and myokines

Myogenic cell proliferation and differentiation are tightly regulated by a complex cascade of growth factors and cytokines, including insulin-like growth factors (IGF-1 and IGF-2), fibroblast growth factors (FGFs), transforming growth factor-beta (TGF-β), platelet-derived growth factor (PDGF), and hepatocyte growth factor (HGF). IGF-1 plays a crucial role in promoting MuSC growth and protein synthesis, whereas FGFs promote MuSC self-renewal and proliferation while generally delaying premature differentiation. Specifically, FGF-2 is critical for maintaining the proliferation of bovine and porcine MuSCs as well as C2C12 myoblasts (Pawlikowski et al., 2017; Ahmad et al., 2020). PDGF promotes myoblast expansion, HGF activates quiescent MuSCs, and TGF-β1 participates in muscle repair and ECM remodeling but can inhibit myogenic proliferation and differentiation depending on the cellular and temporal context (Ahmad et al. 2023b). Additionally, insulin-like growth factor binding proteins 3 and 5 (IGFBP-3 and IGFBP-5) mediate the anti-proliferative effects of TGF-β and myostatin (MSTN) in myogenic cultures (Dayton & White, 2008). Coordinated supplementation with FGFs, IGFs, and PDGF can thus support the in vitro expansion and maintenance of stemness in myogenic populations, although responses vary based on dosage, cell type, and background media composition. Representative studies detailing specific in vitro concentrations, cell models, and functional outcomes of these factors are summarized in Table 2.

**Table 2:** Experimentally reported in vitro concentrations and biological effects of selected growth factors and cytokines in myogenic cell models

Evidence level	Cell type/model	Factor/ cytokine	Reported in vitro dose (ng/mL)	Culture context	Main reported effect	Reference
Tier– Livestock MuSCs	Bovine MuSCs	FGF-2	2	Proliferation	Promoted bovine MuSC proliferation in serum-free medium	Therkildsen et al. (2023)
Tier 1 – Livestock MuSCs	Bovine MuSCs	FGF-2	2; 17.5–70*	Proliferation	Bovine FGF-2 promoted proliferation at a lower concentration than salmon/cod FGF-2	Skrivergaard et al. (2025)
Tier 1 – Livestock MuSCs	Bovine MuSCs	IL-6	0.01	Proliferation	Evaluated as a medium supplement; IL-6 alone did not significantly improve proliferation over the relevant control	Stout et al. (2022)
Tier 1 – Livestock myogenic cells	Primary fetal bovine skeletal myogenic cultures	IL-15	10, 100	Differentiated/ anabolic phase	Increased myosin heavy chain (MYH) accumulation; did not increase proliferation or differentiation rate	Quinn et al. (1995)
Tier 2 – Avian myogenic c1 ells	Chicken embryonic myoblasts	IGF-1	10, 100, 1,000	Proliferation	Dose-dependent promotion of myoblast proliferation through PI3K/Akt-related signaling	Yu et al. (2015)
Tier 2/3 – Rodent and human myogenic cells	C2C12 and human skeletal muscle myoblasts	HGF	2, 10	Proliferation/differentiation	HGF produced dose-dependent effects; 2 ng/mL promoted cell division but reduced myogenic commitment and fusion	Walker et al. (2015)
Tier 2 – Rodent satellite-cell model	Mouse satellite cells associated with single myofibers	SDF-1 (CXCL12)	10	Proliferation/regeneration	Increased the proportion of proliferating satellite cells and accelerated myogenic progression	Brzoska et al. (2012)

The concentrations shown represent experimentally reported concentrations used in the cited studies and should not be interpreted as standardized or optimal doses. Biological responses may vary depending on species, cell type, concentration, exposure duration, and culture phase. Tier 1 represents direct evidence from livestock MuSCs or primary livestock myogenic cells, whereas Tier 2 and Tier 3 represent evidence from non-livestock and human myogenic models, respectively. Concentrations reported in non-livestock models should not be directly extrapolated to cultured-meat MuSC cultures without further validation

Beyond classic growth factors, muscle physiology is strongly modulated by myokines and immune-derived cytokines such as interleukin-6 (IL-6), leukemia inhibitory factor (LIF), IL-4, IL-15, and IL-1β (Shaikh et al., 2021; Pedersen et al., 2007). LIF, an IL-6 family cytokine released during muscle repair and physical activity, promotes myoblast proliferation and contributes to muscle regeneration through JAK/STAT signaling, whereas its effects on differentiation are context- and stage-dependent (Spangenburg & Booth, 2006; Yang et al., 2009; Broholm et al., 2011). IL-15 promotes protein accumulation and myogenic maturation, while enhancing the expression of myogenic markers and myosin heavy chain (MYH) accumulation (Furmanczyk & Quinn, 2003; O’leary et al., 2017). Conversely, IL-6 exerts context- and concentration-dependent effects on myogenesis, with distinct signaling outcomes during proliferation and differentiation (Steyn et al., 2019). Knockdown of STAT3 or IL-6 in C2C12 myoblasts inhibits MYOG and MYH IIb expression and impairs myotube fusion (Hoene et al., 2013). Supplementation with IL-6 has also been shown to significantly enhance bovine satellite cell proliferation in serum-free media (Stout et al., 2022). Furthermore, IL-4 promotes secondary myoblast fusion during myofiber formation (Lafreniere et al., 2006; Chang et al., 2019), and IL-12 enhances C2C12 myogenic progression (Romanazzo et al., 2015).

Contraction-induced myokines further contribute to muscle metabolic homeostasis and anabolic signaling. Myonectin promotes fatty acid uptake in hepatocytes and adipocytes and can suppress hepatocyte autophagy through PI3K/Akt/mTOR signaling, thereby contributing to systemic metabolic homeostasis (Seldin & Wong, 2012; Seldin et al., 2013). Decorin, an activity-dependent proteoglycan present in embryonic and adult SKM, promotes myogenesis, at least in part, by binding and sequestering MSTN in the extracellular matrix (El Shafey et al., 2016; Nishimura et al., 2002). Overexpression of decorin upregulates pro-myogenic genes (Mighty, Myod1, and follistatin) while downregulating muscle atrophy markers (Atrogin1 and MuRF1) (Kanzleiter et al., 2014). Similarly, irisin, a cleavage product of fibronectin type III domain-containing protein 5 (FNDC5), acts through autocrine signaling to regulate muscle homeostasis and tissue repair (Colaianni et al., 2017).

Notably, many studies investigating these regulatory factors have focused on human and rodent models, with comparatively fewer studies addressing livestock-derived MuSCs relevant to cultured meat production. As highlighted in Table 2, findings from C2C12 cells and rodent satellite cells provide valuable mechanistic insights, but the reported effective concentrations and cellular responses vary across species and culture contexts, underscoring the need for livestock-specific optimization. Systematic, species-specific optimization of growth factor and cytokine cocktails tailored to each culture phase, together with the elimination of FBS dependence, therefore remains a major bottleneck for the development of scalable MuSC-based cultured meat production.

### ECM proteins

ECM proteins are essential for maintaining structural integrity and facilitating cell migration, adhesion, proliferation, and differentiation. The ECM in SKM consists primarily of structural and adhesion proteins, including collagens, laminins, and fibronectins (FNs), which provide mechanical support and produce biochemical signals required for myogenesis and tissue repair (Frantz et al., 2010; Ahmad et al., 2018; Loreti & Sacco, 2022). Specific ECM proteins regulate cell–matrix interactions and matrix assembly, dictating the biomechanical and biochemical properties of the muscle microenvironment (Lieber & Meyer, 2023).

Collagen is the most abundant structural protein in the ECM, with multiple collagen types identified in SKM. Type VI collagen is widely distributed throughout many tissues, interacting with ECM components, integrins, and signaling molecules to promote cellular growth, differentiation, and tissue repair (Yokomizo-Goto et al., 2024). Produced primarily by interstitial fibroblasts and concentrated within the interstitial ECM of muscles, type VI collagen is rapidly upregulated following muscle injury; its genetic deletion reduces the number of MuSCs and impairs their activity and regenerative capacity (Urciuolo et al., 2013). Laminin, another important ECM protein, mediates cell adhesion while robustly stimulating MuSC expansion and subsequent myogenic progression (Kim et al. 2024a). Similarly, FN exhibits a high affinity for growth factors such as FGF2 and vascular endothelial growth factor (VEGF). It is highly expressed in damaged muscle tissues, where it activates MuSCs and supports their differentiation into muscle cells (Loreti & Sacco, 2022). Furthermore, FN promotes the fusion and linear alignment of myotubes during differentiation, and its absence is associated with impaired myotube formation and SKM dysfunction (Zhang et al., 2021).

Despite these critical physiological roles, research on ECM protein signaling in livestock-derived MuSCs remains limited. By providing essential microenvironmental cues that coordinate growth factor signaling and cell adhesion molecules, bioengineered ECM interfaces can enhance MuSC proliferation, differentiation, and tissue organization, while potentially lowering serum requirements. However, defining the optimal composition, physiological concentrations, and application methods for specific ECM components remains a key challenge. Furthermore, the commercial deployment of ECM proteins in cultured meat bioprocessing must navigate food-grade regulatory frameworks and cost constraints across global jurisdictions. Resolving these knowledge gaps is essential for developing scalable, reproducible, and cost-effective MuSC cultivation systems.

### MSTN

MSTN, a member of the TGF-β superfamily, is a potent negative regulator of muscle growth, acting primarily by inhibiting myogenic signaling and promoting protein turnover in MuSCs. Genetic disruption of MSTN, as demonstrated in MSTN-null mice and double-muscled cattle, results in marked increases in muscle mass, establishing MSTN as a key target for enhancing muscle development in both livestock and cultured meat production (Mcpherron and Lee, 1997; Mcpherron et al., 1997). MSTN binds to activin type II receptors (ACVR2A/B), leading to activation of SMAD2/3 signaling and suppression of PI3K/Akt signaling. Reduced Akt activity can promote FOXO activation and the expression of muscle atrophy-related genes such as Atrogin-1, thereby contributing to the inhibition of myogenesis. In contrast, IGF-1/PI3K/Akt signaling can antagonize these inhibitory effects, promoting muscle growth (Lin et al., 2021; Sartori et al., 2021). Additionally, fibromodulin (FMOD), an ECM proteoglycan, modulates MSTN activity and supports MuSC proliferation and differentiation through reciprocal regulation, further influencing muscle regeneration and ECM assembly (Lee et al., 2021).

Inhibiting MSTN, ACVR2B, or SMAD2/3 through genetic engineering or natural compounds can enhance MuSC differentiation, promote myotube formation and maturation, and upregulate key myogenic markers such as MYOD, myogenin (MYOG), and MYH (Ahmad et al. 2024c; Wetzlich et al. 2025). Improved myotube development can translate to superior structural and textural attributes in cultured meat, although additional factors such as intramuscular fat content remain important for optimizing flavor and consumer appeal (Guan et al., 2023; Jin & Bao, 2024).

Although other signaling pathways such as Notch, Wnt, metabolic regulation, and cellular senescence also govern MuSC maintenance and lineage commitment, MSTN modulation is emphasized because of its direct influence on myofiber formation and overall tissue yield. Synergistic regulation of MSTN with these complementary pathways can optimize MuSC expansion and differentiation, thus enhancing both productivity and functional quality of cultured meat. However, most MSTN targeted studies have focused on model organisms, with limited data for livestock-derived MuSCs relevant to commercial applications. Critical knowledge gaps remain regarding optimal inhibition strategies and the development of safe, scalable compounds appropriate for industrial bioprocessing. Addressing these challenges is essential for translating MSTN modulation into efficient, reproducible, and regulatory-compliant cultured meat production (Mcpherron and Lee, 1997; Ahmad et al. 2024c).

## Culture technology of MuSCs

### MuSC isolation, cell culture, and storage

Efficient isolation and expansion of MuSCs are foundational to cost-effective cultured meat production. The initial isolation process involves removing extraneous fat and connective tissue from muscle, followed by mechanical mincing and enzymatic dissociation using proteases such as trypsin, pronase, collagenase, or dispase. The choice of enzyme, duration of digestion, and protocol optimization are all critical, as they directly influence cell recovery, viability, and downstream performance. Recent studies show that recombinant proteases and species-specific enzymes can improve yield and minimize the introduction of impurities or contaminants (Lee et al., 2007; Lim et al., 2026). Enzymatic dissociation generates a heterogeneous cell suspension, which is further refined through filtration and centrifugation to remove debris, producing a starting population for MuSC enrichment (Lee et al. 2024c).

Enrichment of MuSCs from the mixed cell suspension is achieved using techniques such as pre-plating, density-gradient centrifugation, fluorescence-activated cell sorting (FACS), and magnetic-activated cell sorting (MACS). FACS and MACS utilize species- and tissue-specific surface markers for precise selection, with FACS offering high purity but limited scalability and higher complexity, whereas MACS provides a more streamlined, scalable approach but requires validated antibodies. Pre-plating and density-gradient methods forgo antibody use, relying on differences in cell adhesion and density, and are simpler but less specific. Method selection should consider not just purity, but also yield, viability, throughput, cost, and compatibility with food-grade bioprocesses (Boscolo Sesillo et al. 2020; Choi et al. 2021; Kim et al. 2022; Lee et al. 2024c).

The optimal isolation strategy must balance cell yield, purity, viability, processing complexity, cost, scalability, and species-specific compatibility with downstream applications (Table 3). High-purity methods such as FACS are valuable for research and cell characterization, but may introduce processing complexity and cell loss that hinder large-scale manufacturing. Simpler approaches like pre-plating or density gradients are better suited for scale-up, provided they yield MuSCs of sufficient quality for expansion and differentiation (Lee et al. 2024b). MACS offers a potential intermediate solution, combining marker specificity with streamlined processing. Ultimately, standardization and head-to-head comparisons across livestock species are needed to define best practices. Protocols should prioritize not only high recovery and initial cell quality but also aseptic processing, minimal antibiotic use, and seamless integration with scalable, closed bioprocessing systems.

**Table 3:** Comparison of MuSC isolation methods and their potential applicability to cultured meat production

Isolation method	Cell yield	Purity	Cell viability	Processing complexity/time	Cost	Scalability potential	Antibody dependence	Species specificity	Potential applicability to cultured meat	Representative reference
Pre-plating	Method-dependent	Moderate; optimization-dependent	Method-dependent	Relatively simple	Relatively Low	Potentially favorable	No	Low	Simple and economical enrichment; suitable when extremely high purity is not required	Kim et al. (2022); Lee et al. (2024c)
Density-gradient centrifugation	Method-dependent	Moderate to low	Method-dependent	Relatively simple	Relatively Low	Potentially favorable	No	Low	Useful for bulk enrichment but provides limited marker-specific purification	Lee et al. (2024c)
FACS	Recovery-dependent	High	Sorting-condition dependent	Complex; relatively time-consuming	High	Limited for large-scale application	Yes	High	Useful for high-purity research and characterization; less attractive as the sole industrial isolation step	Boscolo Sesillo et al. (2020); Kim et al. (2024b)
MACS	Marker- and tissue-dependent	Moderate to high	Method-dependent	Simpler than FACS	Moderate	Potentially greater than FACS	Yes	Moderate to high	Potentially useful for scalable enrichment when validated species-specific reagents are available	Kim et al. (2022); Lee et al. (2024c)˒

### MuSC culture

MuSCs are routinely expanded on collagen- or gelatin-coated plates, which help maintain their stemness and promote robust cell adhesion. Proliferation media typically contain elevated serum concentrations often around 20% FBS alongside supplementary FGF2 to stimulate cell division and prevent premature differentiation. Once cultures reach near confluence (approximately 90% surface coverage), cells are transitioned to differentiation media characterized by reduced serum levels (e.g., 2% FBS or horse serum), prompting MuSCs to exit the cell cycle and fuse into multinucleated myotubes (Zammit et al., 2004). The optimal combination and concentration of serum types and supplements vary by species and are critical for maximizing proliferation and myogenic differentiation efficiency.

For scalable production, MuSCs must be cultured in specialized bioreactors rather than traditional static plates. While bioreactors used in biopharmaceutical manufacturing provide a foundation, cultured meat production demands custom reactor designs that support both MuSC expansion and efficient differentiation into structured myotubes. This necessitates innovations in bioreactor engineering, including dynamic culture conditions, spatial organization, and real-time monitoring. Industry leaders are developing reactors specifically tailored for MuSC cultivation, which differ substantially from those optimized for non-anchorage-dependent cell lines in biopharmaceutical production (Lee et al. 2024a).

### MuSC storage

A stable and reproducible supply of MuSCs is fundamental for continuous cultured meat production, making effective cryopreservation and cell banking essential. Dimethyl sulfoxide (DMSO) is the most widely used cryoprotectant, but its cytotoxicity has prompted the development of DMSO-reduced or DMSO-free protocols (Awan et al., 2020; Weng & Beauchesne, 2020). FBS, traditionally included in cryopreservation media, presents challenges due to batch-to-batch variability and animal-origin components, which are incompatible with fully animal-component-free systems (Liu et al., 2023; Weber et al., 2025). Alternatives such as trehalose offer promise, yet require further optimization for MuSCs due to limited membrane permeability and insufficient livestock-specific data (Murray et al., 2024). Cryopreservation outcomes depend not only on the choice of protectant, but also on exposure time, cooling and warming rates, and other freeze–thaw conditions (Murray & Gibson, 2022). Importantly, the evaluation of cryopreserved MuSCs should extend beyond post-thaw viability to include recovery, proliferative capacity, and myogenic differentiation potential. While studies show that MuSCs can retain myogenic properties after long-term storage, comprehensive species-specific optimization is still lacking (Shigemura et al., 2011). Industrial-scale cultured meat production will benefit from robust, well-characterized cell banks and validated preservation protocols tailored to each livestock species (Mia et al., 2026).

## Challenges and alternatives in MuSC culture media

Culture media for MuSC cultivation are comprised of basal nutrient solutions supplemented with serum, growth factors, hormones, and other bioactive components to support cell proliferation and differentiation. Selecting an appropriate basal medium such as minimum essential medium (MEM), Dulbecco’s modified Eagle medium (DMEM), or Ham’s F12 requires careful consideration of nutrient composition and the specific requirements of the MuSC source and intended application (Yao & Asayama, 2017).

Optimal media formulations for MuSC proliferation and differentiation are highly species-dependent, reflecting distinct biological characteristics and requirements among livestock. Variations in the composition of basal media, amino acids, vitamins, carbohydrates, growth factors, and serum-free supplements can significantly affect proliferation and myogenic differentiation efficiency (Olenic et al. 2025b). Furthermore, culture media must be modulated stage-specifically: expansion media maintain progenitor states, while differentiation media promote myotube formation (Flees et al., 2022; Messmer et al., 2022). Table 4 summarizes representative serum-free media formulations, their compositions, and functional outcomes reported for different species, cell types, and culture stages. This underscores the need for species-, cell type-, and stage-specific media optimization, as media designed for other cell types or culture phases may not be directly applicable to all stages of cultured meat production.

**Table 4:** Representative serum-free media formulations for muscle cell expansion and differentiation in cultured meat production

Medium	Key composition	Target species/cell	Culture stage	Effects on cell proliferation and differentiation	References
Serum-free medium (SFM)	DMEM/F12; BSA 5 mg/mL; HEPES 20 mM; penicillin/streptomycin 1% (v/v); hydrocortisone	Larimichthys crocea myoblasts	Expansion and differentiation	Supported cell attachment and expansion; viable-cell yield was approximately 65% of the serum-containing medium; serum-free culture showed a higher fusion index than serum controls.	Zhang et al. (2024)
Chemically defined serum-free differentiation medium (SFDM)	DMEM/F12 (1:1); EGF 10 ng/mL; human albumin 0.5 mg/mL; 2-phospho-L-ascorbic acid 40 µM; sodium selenite 80 nM; NaHCO3 6.5 mM; MEM amino acids 0.5%; insulin 1.8 µM; transferrin 135 nM; lysophosphatidic acid 1 µM; acetylcholine 10 µM	Primary bovine MuSC	Differentiation	Induced serum-free myogenic differentiation without serum starvation; differentiation was comparable to serum-starvation controls and supported formation of 3D bioartificial muscle.	Messmer et al. (2022)
Commercial serum-free media	FBM, Essential 8, TeSR-E8, and other commercial formulations; exact compositions proprietary/not disclosed	Primary bovine myoblasts	Expansion	Supported proliferation, but cell expansion was generally lower than in serum-containing controls; performance differed among formulations.	Kolkmann et al. (2020)
Beefy-9 serum-free medium	DMEM/F12 (1:1); insulin 20 µg/mL; 2-phospho-L-ascorbic acid 200 µg/mL; transferrin 20 µg/mL; sodium selenite 20 ng/mL; FGF-2 40 ng/mL; TGF-β3 0.1 ng/mL; NRG1 0.1 ng/mL; recombinant human albumin 800 µg/mL; HEPES; antibiotic/antimycotic	Primary bovine MuSC	Expansion	Sustained expansion for seven passages with an average doubling time of 39 h while maintaining myogenicity.	Stout et al. (2022)
Microalgae-derived serum-free conditioned medium (CVNM-CM)	Inorganic salt solution formulated to DMEM-like conditions; Chlorella vulgaris extract (CVE) as nutrient source; RL34-cell conditioned medium containing cell-secreted growth factors; optimized bovine-myoblast condition contained 10% CVE	Primary bovine myoblasts	Expansion	Supported proliferation of bovine myoblasts under serum-free and grain-derived-nutrient-free conditions; growth was lower than in basal medium containing 10% FBS; myogenic identity was maintained.	Yamanaka et al. (2023)
Recombinant-albumin-supplemented serum-free medium	Bovine or porcine recombinant albumin produced in Pichia pastoris; evaluated as a serum substitute in bovine MuSC culture	Primary bovine MuSCs	Expansion and differentiation	Supported bMuSC proliferation and maintenance of satellite-cell characteristics; recombinant albumin also supported myotube formation and maintained differentiation potential.	Kim et al. (2025b)
Serum-free medium for long-term expansion and 3D culture	C2C12 serum-free medium optimized by Plackett–Burman design; key components included bFGF, dexamethasone, insulin, BSA-V, transferrin, palmitoyl tripeptide-5, glutathione, vitamins C/E, folic acid, biotin, cholesterol/lipid components, and ethanolamine	C2C12 myoblasts (mouse)	Expansion and 3D culture	Supported short-term proliferation and long-term passage while maintaining myogenic differentiation potential; microcarrier culture achieved 43.55-fold expansion after seven days.	Dai et al. (2024)

A major challenge in MuSC culture is minimizing or eliminating the use of FBS, which is costly, compositionally variable, and raises ethical and safety concerns. FBS contains an array of hormones, growth factors, and nutrients that support cell growth and metabolism but varies by source and batch, which can affect reproducibility (Van Der Valk et al., 2010; Vultaggio-Poma et al., 2024). Sex- and age-specific differences in adult bovine serum composition further influence MuSC proliferation and differentiation outcomes (Lee et al., 2007).

To address these limitations, various serum-free media and chemically defined media have been developed. These include recombinant protein supplements, plant- or microalgae-derived components, and growth factor cocktails tailored to MuSC requirements. For example, Beefy-9 medium has supported sustained expansion of bovine satellite cells for multiple passages while maintaining myogenicity, illustrating the potential of serum-free approaches (Stout et al., 2022). The use of adult bovine serum as an interim lower-cost alternative remains controversial, and any animal-derived ingredient requires strict quality-control protocols to minimize contamination risks (Lee et al., 2022). Ultimately, the development of robust, cost-effective, and well-characterized serum-free media remains a central goal for the cultured meat industry.

Media compositions must be optimized not only for biological performance but also for industrial scalability and cost-effectiveness. In addition to supporting cell growth and differentiation, media formulations for food-oriented applications must be evaluated with respect to component safety, compositional consistency, and suitability for the intended production process. The osmotic balance of culture solutions must also be carefully maintained (Takagi et al., 2000). Reducing or replacing antibiotics with natural antimicrobial agents remains a technical hurdle in large-scale processes. Media costs represent a significant share of overall cultured meat production expenses; therefore, economic viability as well as biological efficacy must guide the selection and optimization of media components (O'neill et al., 2021).

## In silico approaches

Cultured meat production shares technological similarities with biopharmaceutical manufacturing, but it uniquely requires that all cell culture components be safe and suitable for human consumption. Thus, the safety and appropriateness of media additives including animal serum, recombinant growth factors, hormones, cytokines, and antibiotics must be carefully assessed for their use in cultured meat production.

In silico approaches, including computational screening and AI-assisted methods, are valuable for prioritizing candidate compounds from large natural-product databases such as COCONUT, LOTUS, and ZINC (Irwin et al., 2020; Sorokina et al., 2021; Rutz et al., 2022). These methods can predict interactions between candidate molecules and biological targets relevant to MuSC proliferation and differentiation, but predictions require substantial experimental validation to confirm suitability for cultured meat applications. For instance, docking and molecular modeling have identified natural compounds such as licochalcone A and B, quercetin, and laxogenin as potential MSTN inhibitors (Ahmad et al. 2024a, c; Lim et al., 2025), providing a rational basis for experimental testing rather than definitive evidence of efficacy.

Experimental follow-up has shown that selected compounds, including licochalcone A & B (Ahmad et al. 2024a) and laxogenin (Lim et al., 2025) can enhance MuSC proliferation and differentiation, while quercetin offers antioxidant protection during culture, mitigating oxidative stress and improving cell viability (Ahmad et al. 2024c). These studies demonstrate the practical value of in silico screening for hypothesis generation and initial compound selection. However, most findings are derived from muscle biology or regeneration models, and their direct relevance to cultured meat production in livestock MuSCs, including effects on tissue formation, product quality, and scalability, remains to be fully established.

An important goal for cultured meat medium formulation is to develop alternatives to conventional antibiotics, which are used to prevent microbial contamination but can induce cellular toxicity and alter gene expression (Ryu et al., 2017; Rivetti et al., 2023). In silico screening can help identify candidate antimicrobial compounds from large chemical libraries, but their antimicrobial efficacy, cytotoxicity, residual levels, food safety, and regulatory suitability must be experimentally evaluated before their use in food-oriented cell culture systems. The use of computational methods could accelerate the development of antibiotic-free or reduced-antibiotic culture systems if confirmed effective in food-relevant cell types.

AI and in silico approaches can facilitate cell quality assessment, medium optimization, metabolic analysis, and process development in cultured meat research (Table 5). Multi-objective AI-based optimization has been used to design low-serum media with reduced environmental impact and cost for zebrafish cells (Nikkhah et al., 2023), and Bayesian optimization has streamlined serum-free medium development for C2C12 myoblasts (Cosenza et al., 2022, 2023). Image-based prediction and machine learning have been applied to assess porcine and Hanwoo MuSC quality and senescence (Wang et al. 2023a, b; Park et al. 2026). Although these studies demonstrate the feasibility and promise of computational approaches, further validation in primary livestock MuSCs and bioprocess-scale systems is required to translate these findings into commercial cultured meat production.

**Table 5:** Applications of AI and computational approaches in cultured meat production

Application of AI and computational approaches	Function and representative evidence
Cell Quality and Potency Assessment	Image-based computational approaches can quantify cellular morphology and support the prediction of proliferation and differentiation potential. In porcine MuSCs, morphological features were used to predict proliferation and differentiation potency, demonstrating the potential of computational approaches for cell-quality assessment in cultured meat production (Wang et al. 2023a, b).
Cell Quality Control	Machine-learning approaches can integrate multiple cellular characteristics to assess passage-dependent cell quality. In Hanwoo MuSCs, machine learning was used to integrate proliferation- and senescence-related biomarkers and classify cellular quality across passages, providing a potential framework for quality control in cultured meat production (Park et al., 2026).
Media Formulation	Bayesian optimization can efficiently identify culture-medium formulations by modeling the relationships among medium composition and cell growth. In a cellular-agriculture model using murine C2C12 myoblasts, a 14-component medium was optimized using Bayesian optimization, resulting in 181% more cells than a commercial medium with fewer experiments (Cosenza et al., 2022). A subsequent study used multi-objective Bayesian optimization to simultaneously improve serum-free C2C12 cell growth and reduce medium cost (Cosenza et al., 2023).
Metabolic and Bioprocess Optimization	Systems biology and metabolic modeling can characterize cell-specific metabolic requirements and identify potential metabolic bottlenecks relevant to culture-medium and bioprocess optimization in cultivated meat production (Gomez Romero & Boyle, 2023).
Process and Scale-up Optimization	Computational modeling may support the evaluation of nutrient requirements and culture-process parameters during process development and scale-up. However, direct experimental validation of AI-based industrial-scale applications remains limited (Gomez Romero & Boyle, 2023).

**Fig. 2:**
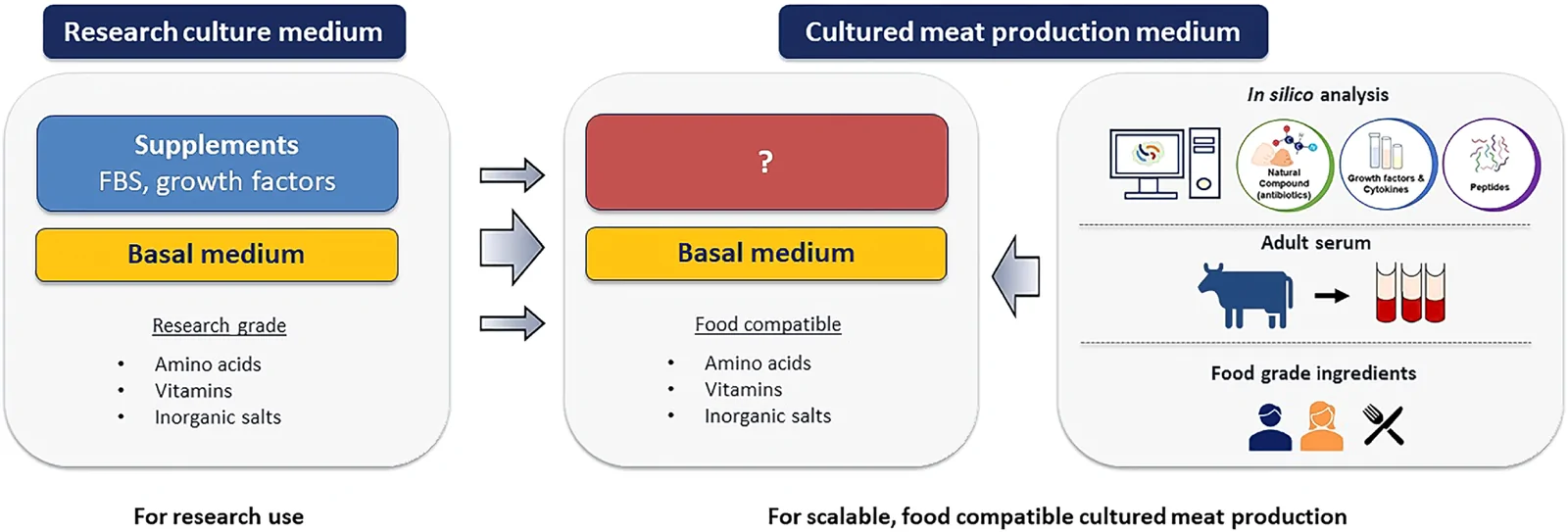
Media composition for cell culture and cultured meat production. Illustrates the transition from standard basal media and supplements in cell culture to food-grade ingredients for cultured meat production. Depicts the integration of in silico and AI analysis to identify or optimize culture media composition for industrial-scale applications

When interpreting computational predictions for MuSC-based cultured meat applications, several technical factors must be considered, including docking score reliability, protein structure quality, and the need for experimental confirmation of solubility, cytotoxicity, and effective concentration in food-relevant cells, particularly when homology models are used. Candidate compounds should also be experimentally evaluated for solubility, cytotoxicity, and effective concentrations in food-relevant cells. Promising compounds should be tested in 3D and bioreactor-scale cultures to assess their process-relevant effects. As such, in silico and AI approaches should be regarded as complementary to, rather than replacements for, rigorous experimental optimization (Fig. 2).

## Scaffolds for cultured meat

Scaffolds provide essential platforms for cell attachment, proliferation, organization, and differentiation and are critical for engineering 3D cultured meat structures. For MuSC-based cultured meat, scaffold design should be tailored to the unique requirements of MuSC biology, including optimal cell adhesion, proliferation, myogenic differentiation, and cellular alignment, rather than relying solely on general tissue engineering principles. ECM derived or ECM-mimetic scaffolds recreate a supportive microenvironment, promoting the assembly of MuSCs into organized, muscle-like tissues (Lee et al. 2016; Ahmad et al. 2021, 2024b).

Scaffold materials for cultured meat production are typically classified as natural, synthetic, or composite. Animal derived collagen and gelatin remain prevalent, but concerns about sourcing and consumer acceptance have driven the exploration of plant-based alternatives such as cellulose, pectin, alginate, and novel edible proteins (Seah et al., 2022). Recent advances include soy protein based porous scaffolds and composite scaffolds using whey protein isolate, methacrylated alginate, and visible light crosslinking (Wei et al., 2023; Charron et al., 2024). However, while many scaffold studies utilize C2C12 myoblasts, relatively few have directly investigated primary livestock MuSCs. Noteworthy examples include bovine satellite cells cultured on zein-coated alginate fibers and decellularized mushroom-derived scaffolds. Table 6 summarizes representative scaffold materials, architectures, and applications for cultured meat and SKM tissue engineering.

**Table 6:** Representative scaffold biomaterials for muscle tissue engineering and cultured meat production

Scaffold Type	Material/Structure	Cell Model	Application	Key Findings	References
Zein-coated alginate fiber	Plant-derived, stretchable zein-coated alginate microfiber	Bovine satellite cells with adipocytes and endothelial cells	Cultivated-meat fiber alignment and structuring	Supported cell adhesion and proliferation; mechanical stretching promoted muscle-cell alignment and myotube maturation; muscle, fat, and vascular fibers were assembled into a cultivated-meat construct	Jeong et al. (2024)
Decellularized *Pleurotus ferulae* dECM scaffold	Decellularized edible mushroom-derived ECM with a fibrous microarchitecture	Bovine MuSC	Steak-like cultured-meat scaffold	Supported bovine MuSC adhesion and proliferation and promoted aligned differentiation into parallel myotubes; engineered constructs exhibited meat-like structural and textural characteristics	Kim et al. (2025a)
Decellularized parsley scaffold	Gelatin/transglutaminase-modified decellularized parsley with longitudinal fibrous or transverse honeycomb pores	C2C12 myoblasts	Cultured-meat scaffold	Fibrous pore structures guided cell alignment and enhanced myogenic differentiation; fibrous scaffolds showed increased myogenic gene/protein expression and protein content in the resulting constructs	Chen et al. (2024)
Decellularized celery scaffold	Plant-derived decellularized celery with a porous native architecture	Chicken myoblasts	Chicken skeletal muscle tissue formation	Supported chicken myoblast proliferation and differentiation; cells covered the scaffold surface and formed fiber-like myotube structures, supporting skeletal muscle tissue formation	Hong and Do (2024)
Electrospun PCL/GelMA hybrid scaffold	Aligned electrospun PCL fibers incorporated into a GelMA hydrogel	Muscle-derived cells	Skeletal muscle tissue engineering	Aligned PCL fibers improved mechanical properties, cell alignment, viability, and proliferation; the hybrid scaffold promoted MYH2 expression and myotube formation	Hong et al. (2025)
Decellularized asparagus plant scaffold	Decellularized asparagus with aligned vascular bundles and a macroporous structure	C2C12 myoblasts and porcine adipose-derived MSCs	Cultivated-meat muscle/fat tissue engineering	Vascular bundles promoted C2C12 attachment and alignment and supported myogenic differentiation; co-culture with porcine adipose-derived MSCs enabled generation of a muscle–fat cultured-meat prototype	Murugan et al. (2024)
Scaffold biomaterials (review)	Animal-, plant-, algae-, and microbial-derived biomaterials	Multiple cell models	Cultured-meat scaffold design and selection	Scaffold biomaterials influence cell adhesion, proliferation, differentiation, and tissue structure; plant-derived scaffolds offer potential advantages in edibility, biodegradability, and cost, while mechanical strength and biocompatibility remain important considerations	Park et al. (2025)

Beyond physical support, scaffolds modulate MuSC behavior through cell-matrix and cell-cell interactions. ECM-related gene expression changes and immunoglobulin-like cell adhesion molecules have been implicated in MuSC proliferation, differentiation, and communication (Lee et al. 2012, 2016; Lim et al. 2021, 2022; Ahmad et al. 2023a). While these molecular insights provide valuable guidance, they do not prescribe specific scaffold architectures; instead, they suggest that designs mimicking muscle bundle organization, supporting cell alignment and facilitating communication, may enhance myogenic outcomes (Lim et al., 2022; Jeong et al., 2024; Yao et al., 2024). Scaffold selection for food applications must also consider edibility, biocompatibility, porosity, nutrient diffusion, mechanical strength, scalability, and cost (Ong et al., 2021). Importantly, optimal scaffold requirements may differ between expansion and differentiation stages, necessitating evaluation of scaffold materials in the context of their effects on MuSC growth and maturation throughout the culture process.

## Engineering MuSCs for scalable cultured meat applications: bioreactors, microcarriers, and biomimetic niches

The industrial-scale production of cultured meat using MuSCs requires the development of robust bioprocess systems capable of supporting high-density cell expansion while preserving myogenic potential. MuSCs are anchorage-dependent adult stem cells, necessitating close interaction with ECM components to maintain their proliferative and differentiation capacity during in vitro culture (Antebi et al., 2015). Scalable expansion is often achieved using microcarrier-based culture in dynamically mixed bioreactors, where microcarriers provide an increased surface area for cell attachment and growth. These systems have emerged as a promising strategy for industrial-scale muscle cell expansion, with their performance determined by microcarrier material, cell attachment efficiency, microcarrier concentration, and hydrodynamic conditions (Verbruggen et al., 2018; Tzimorotas et al., 2023; Alizadeh Sardroud et al., 2025).

Operational parameters such as cell seeding density, microcarrier concentration, culture duration, and bioreactor volume are critical for evaluating and optimizing scalability. Bodiou et al. (2025) demonstrated successful expansion of bovine satellite cells at microcarrier concentrations up to 80 cm²/mL and seeding densities of 1,000 − 4,750 cells/cm², with process transfer to 3 L bioreactors. Edible microcarriers, such as those based on soy protein isolate, have enabled 24-fold expansion of bovine myoblasts over 8 days, followed by efficient differentiation into myotubes without requiring cell detachment for downstream processing (Geurs et al., 2025). The use of edible or food-grade microcarriers reduces processing complexity and enhances the suitability of the final product for consumption. Porous gelatin-based microcarriers have also shown promise in supporting porcine and bovine MuSC expansion and subsequent myogenic differentiation (Liu et al., 2022; Norris et al., 2022).

Bioreactor operation must balance agitation, oxygenation, nutrient supply, and cell density to support robust cell growth while minimizing hydrodynamic stress. Excessive agitation can reduce cell viability, whereas insufficient mixing limits mass transfer and cell expansion. Optimal microcarrier and bioreactor selection thus requires a holistic approach, considering not only cell expansion and differentiation performance but also cell recovery, harvesting efficiency, material safety, and compatibility with food-grade manufacturing.

Engineering biomimetic microenvironments through scaffold design and matrix composition can help preserve MuSC stemness during large-scale expansion and promote efficient differentiation. ECM composition and physical properties modulate MuSC activation, self-renewal, and fate decisions, indicating the value of biomimetic substrates for controlling stem cell behavior throughout the production process (Thomas et al., 2015). Furthermore, selection of optimal MuSC sources, including consideration of anatomical origin, can influence proliferation rates, differentiation capacity, and protein composition, all of which are relevant to cultured meat yield and quality (Lee et al. 2026b; Xie et al. 2026).

## Scale-up, cost drivers, and life cycle assessment considerations in MuSC-based cultured meat production

Scaling MuSC expansion from laboratory to industrial scales remains a major challenge, despite significant advances in bioreactor and scaffold technologies. Large-scale production demands precise control of cell culture parameters, including oxygenation, pH, nutrient supply, and waste removal, to sustain high cell productivity and consistent cell quality (O’neill et al., 2021, 2022). Scale-up efficiency has important implications for economic feasibility as bioreactor scale, process efficiency, and culture conditions influence the overall cost structure (Negulescu et al., 2023). In some production scenarios, culture media, particularly growth factors and recombinant proteins used in serum-free systems, have been estimated to account for around 50% or more of variable operating costs, although this proportion varies with media formulation, production scale, and underlying assumptions (Quek et al., 2024; Mainali et al., 2025). Scaffold materials, bioreactor operation, energy consumption, and downstream processing further contribute to costs, with their impact varying according to system configuration and process scale. Improving cell productivity, reducing medium requirements, and shortening culture duration are critical for enhancing economic viability (Negulescu et al., 2023; Quek et al., 2024). The development of cost-effective, chemically defined, serum-free media for MuSC expansion is therefore essential to achieving scalable, affordable cultured meat production (O’neill et al., 2021; Quek et al., 2024).

Economic and environmental sustainability are tightly linked in cultured meat production. Process improvements that enhance cell productivity or reduce media consumption can decrease both costs and resource use per unit of biomass. However, these benefits are process- and scale-dependent, and their magnitude is influenced by specific production configurations (Negulescu et al., 2023; Quek et al., 2024). Life cycle assessment (LCA) is crucial for evaluating environmental impacts, as energy use, media ingredient production, and downstream processing can significantly affect the overall environmental footprint of cultured meat (Mattick et al., 2015; Risner et al., 2025).

LCA results for cultured meat are highly sensitive to system boundaries, production scenarios, energy sources, production scale, and other underlying assumptions. Scenario analyses demonstrate that factors such as growth medium purification can dramatically alter environmental impact estimates (Risner et al., 2025). Thus, environmental performance should be evaluated using process-specific assessments, rather than relying on generalized or static LCA values.

Media optimization is a critical intersection of economic and environmental sustainability. Reducing the quantity or cost of growth factors and recombinant proteins, improving nutrient utilization, and developing more sustainable media components can potentially reduce both costs and resource use (Quek et al., 2024; Mainali et al., 2025). Similarly, optimizing bioreactor productivity and scale-up efficiency may reduce environmental impacts per kilogram of product, although benefits depend on the specific process and LCA methodology (Mattick et al., 2015; Negulescu et al., 2023; Risner et al., 2025). Integrating biological performance metrics with techno-economic and LCA analyses will help identify strategies that maximize productivity while minimizing costs and environmental burdens. Ultimately, the success of MuSC-based cultured meat production will depend on holistic approaches that combine bioprocess engineering, microenvironmental optimization, economic analysis, and environmental stewardship.

## Safety concerns for cultured meat production

Cultured meat offers several advantages over conventional meat, but its unique production processes introduce potential food-safety risks that must be systematically assessed prior to commercialization. Hazards may arise at each stage from cell sourcing and expansion to harvesting and formulation and include biological (microbial contamination), chemical (residual antibiotics, growth factors, or reagents), and physical (foreign materials) risks. Prolonged cell culture may also result in genetic or phenotypic alterations, affecting product consistency and reproducibility (Sogore et al., 2024; Zandonadi et al., 2025). However, the mere presence of genetic changes does not automatically equate to a food-safety risk; their relevance must be evaluated in the context of cell biology, process control, and final product characteristics. Comprehensive safety assessment should encompass cell identity, genomic stability, growth and differentiation potential, and product specific safety attributes. Regulatory requirements and testing approaches are evolving globally, necessitating standardized, scientifically justified frameworks for evaluating microbiological safety, residual substances, allergenicity, and nutritional value. Risk-based, process-, and product-specific approaches, supported by preventive measures such as Hazard Analysis and Critical Control Points (HACCP) and GMPs are essential for managing food safety in cultured meat production.

Microbial contamination remains a major concern in cell-based production. While antibiotics may be used at the research scale, routine use in food production is discouraged due to the risk of residues and antimicrobial resistance. Animal-derived components such as FBS introduce additional safety and regulatory challenges, including batch-to-batch variability and biological contaminants. The use of defined, traceable, and well-characterized media components is critical for food applications, and novel ingredients must be appropriately assessed for safety before approval.

Scaffolds intended for cultured meat must be carefully evaluated for composition, biocompatibility, degradation products, and food suitability. For scaffolds remaining in the final product, toxicological and allergenicity assessments are vital. Non-food-grade materials may require additional regulatory review to establish their safety for food use. Scaffold selection should consider not only support for cell growth and tissue formation, but also the safety of scaffold materials and their breakdown products (Ong et al., 2021; Bomkamp et al., 2022).

The origins and characteristics of cell sources are also central to safety assessment. Primary cells have a finite replicative lifespan, whereas intentionally immortalized cells involve genetic or epigenetic modification. Both require comprehensive characterization of identity, genetic and phenotypic stability, and safety attributes prior to use in food production. Media components, including FBS, recombinant proteins, and antibiotics, must be scrutinized for source, purity, toxicological relevance, and food suitability (Andreassen et al., 2020; O’neill et al., 2021).

Regulatory oversight of cultured meat is evolving internationally. Jurisdictions such as Singapore, the United States, and Israel have established regulatory pathways for cultivated animal-cell products, albeit with differing requirements. Singapore classifies cultured meat as a novel food with mandatory pre-market safety assessments, while the US FDA and USDA share oversight responsibilities. These differences underscore the need for science-based, risk-based safety assessment frameworks and continued international regulatory development.

## Perspective and future directions

Cultured meat has the potential to become an important alternative source of animal protein and, under appropriately optimized production scenarios, may reduce land use and certain environmental burdens associated with conventional livestock production. However, the path to successful commercialization is contingent on overcoming a series of interconnected scientific and technological challenges, including cell-source stability, food-compatible and cost-effective culture media, scalable bioprocessing, tissue organization, and robust product safety. Future research should seek integrated solutions that simultaneously address productivity, consistency, safety, and economic feasibility, rather than tackling these challenges in isolation.

This review has summarized the biological and engineering requirements for MuSC-based cultured meat and identified critical research priorities for advancing the field:

1. **MuSC stability and productivity**: Establish robust protocols to maintain MuSC identity, proliferative capacity, and myogenic potential through extended expansion. Standardized assessment of cell identity, genomic stability, differentiation capacity, and batch-to-batch reproducibility should be employed to achieve an optimal balance between expansion and functional maintenance.
2. **Defined**,** food-grade culture media**: Accelerate the development of serum-free, chemically defined, and food-compatible media by identifying essential factors that can substitute for animal-derived components. Evaluation should include not only biological efficacy but also food safety, scalability, and cost-effectiveness.
3. **Scalable bioprocessing**: Translate laboratory-scale protocols to high-density, industrial-scale culture systems by optimizing bioreactor operation, nutrient and oxygen delivery, cell attachment and suspension strategies, and contamination control in tandem with media and process evaluation.
4. **Tissue organization and product quality**: Prioritize research that advances controlled myogenic differentiation, myofiber alignment, ECM organization, and mechanical properties to enhance structure, texture, and nutritional value. Scaffold engineering should be integrated with cell and bioprocess optimization.
5. **AI and in silico approaches**: Leverage AI and computational modeling to prioritize media components, predict cell-material interactions, and optimize culture strategies. Computational findings should guide experimental design and be validated in vitro and at scale.

The integration of stable MuSC sources, well-defined media, scalable bioprocesses, and advanced tissue engineering supplemented by AI and in silico tools offers a practical pathway toward economically viable, safe, and consumer-acceptable cultured meat. Progress should be tracked using quantitative indicators such as productivity, differentiation efficiency, reproducibility, cost, safety, and product quality to enable iterative improvement and industry-relevant benchmarking.

## Data Availability

No datasets were generated or analysed during the current study.
